# A comprehensive assessment of predictors of fertility outcomes in men with non-obstructive azoospermia undergoing microdissection testicular sperm extraction

**DOI:** 10.1186/s12958-020-00646-4

**Published:** 2020-08-26

**Authors:** Parviz K. Kavoussi, Brady T. West, Shu-Hung Chen, Caitlin Hunn, Melissa S. Gilkey, G. Luke Machen, Keikhosrow M. Kavoussi, Amy Esqueda, J. David Wininger, Shahryar K. Kavoussi

**Affiliations:** 1 Austin Fertility & Reproductive Medicine/Westlake IVF, 300 Beardsley Lane, Building B, Suite 200, Austin, TX 78746 USA; 2 Institute for Social Research, University of Michigan, Ann Arbor, MI 48109 USA

**Keywords:** Microdissection testicular sperm extraction, Non-obstructive azoospermia, Pregnancy, Live birth

## Abstract

**Background:**

Microdissection testicular sperm extraction (microTESE) in men with non-obstructive azoospermia (NOA) is the procedure that results in the highest number of sperm cells retrieved for in vitro fertilization (IVF). This study presents a novel assessment of predictors of sperm retrieval as well as downstream embryology and pregnancy outcomes in cases of men with NOA undergoing microTESE.

**Methods:**

A retrospective chart review of 72 men who underwent microTESE for predictors of fertility outcomes including sperm retrieved at microTESE, embryology progression to embryo transfer (ET), clinical pregnancy, live birth, and surplus sperm retrieved for additional IVF/intracytoplasmic injection cycles beyond one initial cycle. Statistical models for each of these outcomes were fitted, with a *p*-value of < 0.05 considered significant for the parameters estimated in each model.

**Results:**

Seventy-two men underwent microTESE, and 51/72 (70.8%) had sperm retrieved. Of those, 29/43 (67.4%) reached ET. Of the couples who underwent ET, 21/29 (72.4%) achieved pregnancy and 18/29 (62.1%) resulted in live birth. Of the men with sperm retrieved, 38/51 (74.5%) had surplus sperm cryopreserved beyond the initial IVF cycle.

Age, testicular volume, FSH, and testicular histopathology were assessed as predictors for sperm retrieved at microTESE, progression to ET, pregnancy, live birth, and surplus sperm. There were no preoperative predictors of sperm retrieval, clinical pregnancy, or live birth. Age predicted reaching ET, with older men having increased odds. FSH level had a negative relationship with surplus sperm retrieved. Men with hypospermatogenesis histology had higher rates of sperm retrieval, clinical pregnancy, live birth, and having surplus sperm.

**Conclusions:**

Men who underwent microTESE with a hypospermatogenesis histopathology had better outcomes, including higher rates of sperm retrieval, clinical pregnancy, live birth, and having surplus sperm retrieved. Increasing male partner age increased the odds of reaching ET. No other clinical factors were predictive for the outcomes considered.

## Background

Approximately 1% of men in the United States and 5–10% of men evaluated for infertility are azoospermic [1, 2]. It has been established that microdissection testicular sperm extraction (microTESE) allows for retrieval of the largest number of sperm cells for use with in-vitro fertilization/intracytoplasmic sperm injection (IVF/ICSI) in men with non-obstructive azoospermia (NOA) with primary testicular dysfunction [3–6]. A number of studies have assessed potential predictors of sperm retrieval rates in men with NOA who underwent microTESE, such as age, follicle stimulating hormone (FSH) level, and testicular volume on sperm retrieval rates [7–20]. However, there is a paucity of data regarding these factors as predictors of downstream outcomes, such as progression of embryology following IVF/ICSI to reach embryo transfer (ET), clinical pregnancy, live birth, and retrieving surplus sperm for more than the initial IVF/ICSI cycle, which can aid in patient management and counseling.

Although sperm retrieval is clearly a rate-limiting first step, the goal of the couple is not simply retrieved sperm but also downstream outcomes, and ultimately live birth. Surplus sperm beyond an initial IVF/ICSI cycle may be important for the couple as well depending on family planning. There has been little data on microTESE outcomes other than sperm retrieval thus far including a study by Yildirim et al. which evaluated sperm retrieval and pregnancy rates and found a negative correlation with FSH levels [21]. Aydin et al. performed a retrospective analysis which revealed that sperm retrieval rates were highest in men with hypospermatogenesis histological patterns in comparison with men who had Sertoli cell only or maturation arrest patterns, but fertilization and pregnancy rates were similar regardless of histology. Live birth rates and surplus sperm retrieval rates were not reported [22]. A meta-analysis published in 2019 assessed sperm retrieval rates as the primary outcome, and IVF outcomes such a pregnancy and live birth as secondary outcomes. This meta-analysis did not report predictors of embryology outcomes, including progressing to ET or having surplus sperm retrieved for more than one IVF/ICSI cycle with microTESE sperm retrieved. In addition, outcomes based on testicular histopathology were not reported [23]. The present study aims to address these gaps in knowledge by identifying predictors of sperm retrieval as well as downstream embryologic and pregnancy outcomes, while accounting for male and female fertility factors.

## Methods

### Patient selection

Between January 2012 and January 2019, men diagnosed with NOA at a couples fertility center, due to primary testicular dysfunction, underwent microTESE (*n* = 72) by a single reproductive urologist (PKK) to be used for IVF/ICSI after having had a minimum of two semen analyses with centrifugation revealing azoospermia, serum hormone testing, and genetic testing with karyotypes and Y chromosome microdeletions assays, the results of which are described in Table 1. The diagnosis of NOA was additionally made by atrophic testicular volumes on physical examination, normal semen volumes, and elevated FSH levels, consistent with primary testicular dysfunction.

**Table 1:** Characteristics of men who underwent microTESE for NOA (*n* = 72) represented in means and standard deviations or percentages

Age (years)	34.1 ± 7.6
Testicular volume (mL)	11.4 ± 6.0
FSH (mIU/mL)	19.5 ± 16.0
Klinefelter Syndrome	9/72 (12.5%)
AZFc Y chromosome microdeletion	4/72 (5.6%)
Bilateral microTESE	35/72 (48.6%)
Had prior attempt at sperm retrieval	8/72 (11.1%)
History of chemotherapy	2/72 (2.8%)
History of cryptorchidism	8/72 (11.1%)
History of testicular torsion	3/72 (4.2%)
Female partner with diminished ovarian reserve	9/64 (14.1%)
Histology of hypospermatogenesis	25/72 (34.7%)
Histology of maturation arrest	7/72 (9.7%)
Histology of Sertoli cell only	40/72 (55.6%)

### MicroTESE technique

MicroTESE was performed with a median raphe incision to allow access to bilateral testes if needed. The larger testis was selected to start and under microsurgical visualization an equatorial incision was made between the subtunical vessels and the entirety of the testicular parenchyma was dissected through under 20x magnification, extracting the fuller appearing seminiferous tubules amidst the sclerotic ones which compromise the majority of the testicular parenchyma.

### Sperm isolation

Initially, sperm is isolated for estimation of successful retrieval and for guiding further microdissection in the operating room under dissecting and inverted light microscopy by the embryologist. An initial mincing is performed with two 27-gauge needles, finely teasing apart the small surgically removed pieces of testicular parenchyma in media. Further extensive mincing is performed post-operatively in the IVF laboratory. At the time of sperm isolation for use, the thawed samples are treated with pentoxifylline and processed in a 15 mL conical tube in MHM + 10% HSA. The sample is plated in microdrops of 8–10 μL of sperm wash media. ICSI pipettes are filled with PVP from a 10 μL drop from the same dish as the microdrops of sperm wash. Two to 3 μL of the treated microTESE sample are put into at least 5 microdrops. Microscopic examination for sperm takes place which can take up to 2 to 3 h. The PVP filled ICSI injection needle is used to pick up the best quality spermatozoa. Once adequate numbers of spermatozoa are isolated for the number of oocytes available, the oocytes are stripped and the ICSI process is started.

### Sperm cryopreservation protocol

After isolation of sperm from the microTESE samples, freeze medium was added to the specimen at a 1:1 dilution, slowly while gently vortexing the sample to mix. The medium was then aliquoted into designated specimen vials. The vials were then suspended on aluminum canes and immersed in a container of room temperature water which was then placed in the refrigerator for 30–90 min. The canes were then quickly transferred to liquid nitrogen vapor for 30–45 min suspended 10–20 cm above the liquid nitrogen surface. The canes were then placed directly into the liquid nitrogen storage tank.

After institutional review board exemption was obtained due to the de-identified nature of the data collected, a retrospective chart review was performed to assess the predictors of outcomes of these 72 men, who underwent microTESE by a single reproductive urologist.

### Variables

The preoperative predictors obtained from the chart review included age, testicular volume, FSH levels, and testicular histopathology with possible categorization of hypospermatogenesis, maturation arrest, or Sertoli cell only pattern. Testicular biopsy was obtained for permanent section histopathology at the time of microTESE, which was read by a pathologist at the operating hospital as well as confirmed by a second pathologist at Mayo clinic. The histopathology was therefore not available preoperatively.

**Fig. 1:**
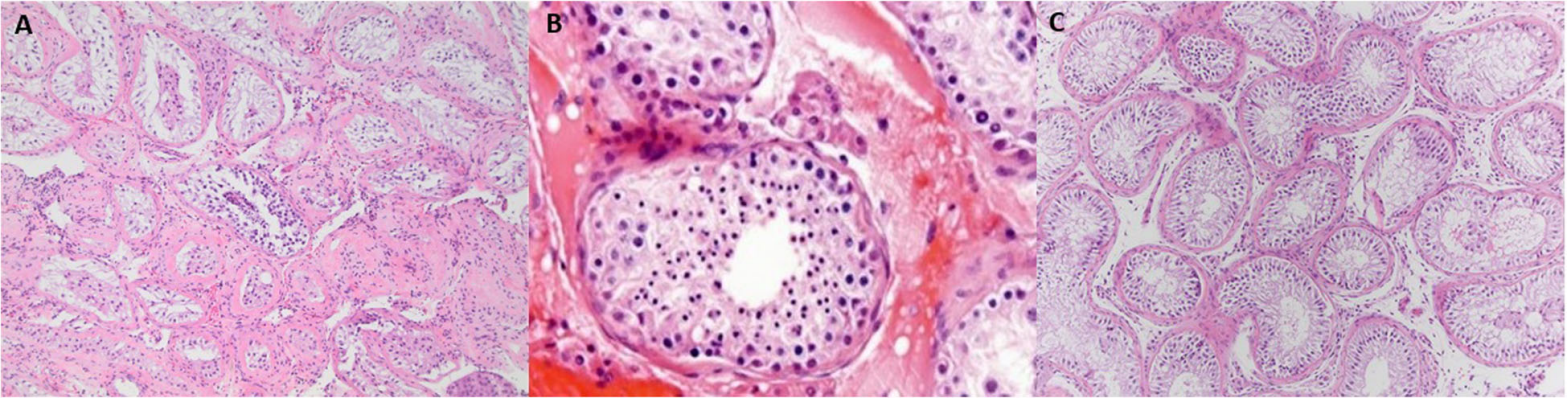
Representative histopathologic images of **a**. hypospermatogenesis patterns, **b**. maturation arrest pattern, **c**. Sertoli cell only pattern from testicular biopsies of men who underwent microTESE for NOA

A hypospermatogenesis pattern was defined as testicular parenchyma with areas of focal severe atrophy, but with evidence of sparse, active spermatogenesis in the non-atrophic tubules. The histopathologic finding of Sertoli cell only and maturation arrest patterns were consistent with standard described pathologic findings of these patterns (Fig. 1). Outcomes assessed for these predictors obtained from the chart review included sperm retrieved at microTESE, progression of embryology to ET after fertilization with ICSI, clinical pregnancy, live birth, and surplus sperm cryopreserved beyond one cycle of IVF/ICSI.

Surplus sperm for future cycles is defined as adequate numbers of cryopreserved sperm in vials maintained beyond the initial IVF/ICSI cycle for use with additional IVF/ICSI cycles based on experienced embryologists assessments of the initial specimens used for the first cycle. This was confirmed by some couples who have undergone multiple IVF/ICSI cycles after sperm retrieved from one microTESE surgery.

### Statistical analysis

We began with simple descriptive analyses to evaluate the distributions of the five outcomes (sperm retrieved at microTESE, progression of embryology to ET, clinical pregnancy, live birth, and surplus sperm cryopreserved beyond one cycle of IVF/ICSI) and the predictors, and describe our analytic sample. Each outcome variable was then modeled using logistic regression analysis, with a given binary indicator (e.g., sperm retrieval) as the outcome, and the factors of interest [male patient age, testicular volume (measured by Prader orchidometer), FSH level, and testicular histopathology], each of which were measured prior to the outcomes being measured, as predictors. Results were described via estimates of adjusted odds ratios and 95% confidence intervals. We could not consider more than four independent predictors given the reduced size of our sample and concerns about reliability of the estimated regression coefficients that would arise from trying to include more predictors [24]. All analyses were performed using the Stata software (Version 16.1; College Station, Texas).

## Results

### Descriptive statistics

The characteristics of the men in our analytic sample are described in Table 1. None of the men in this cohort had children prior to their NOA diagnosis, nor did they have semen analyses with sperm in the semen prior to their diagnosis of azoospermia. Eight of the 72 men (11.1%) had a palpable varicocele on initial presentation. None of the 72 men had a varicocele present at the time of microTESE; therefore, they either never had a varicocele or had it repaired 3 to 6 months prior to microTESE. Of the men who underwent microTESE, 51/72 (70.8%) had sperm at retrieval. The mean operative time for microTESE was 105 min, and one to two embryologists were present in the operating room searching microscopically for retrieved spermatozoa in microTESE specimens while the surgery was being performed. The specimens were then taken back to the IVF laboratory and minced and examined for another 90 to 120 min, were left in media overnight, and reassessed the following morning prior to cryopreservation. All samples of retrieved sperm were cryopreserved for future use. For the MicroTESE cases in which sperm was present and cryopreserved, controlled ovarian stimulation of the female partner was subsequently initiated. All female partners had undergone fertility evaluation and assessment for candidacy for IVF by a reproductive endocrinologist in the same couples fertility center, prior to microTESE. Sperm were thawed for IVF/ICSI on the day of oocyte retrieval. Of the nine men diagnosed with Klinefelter syndrome, seven of them had sperm retrieved (77.8%).

Of the 51 men with sperm retrieved, 29/43 (67.4%) had embryology progress to allow for ET, and the remaining eight either cryopreserved sperm for future use and have not undergone IVF/ICSI yet, or transferred retrieved sperm to another center for IVF/ICSI, where complete data was not available. Of the couples who underwent ET, 9/29 (31%) had day 3 embryo transfers and 20/29 (69%) had day 5 blastocyst transfers, which was at the discretion of the reproductive endocrinologist and embryologist. Of the couples who underwent ET, 21/29 (72.4%) achieved a clinical pregnancy and 18/29 (62.1%) resulted in live birth. Clinical pregnancy was defined as confirmation of a fetal heartbeat, via transvaginal sonogram, at 6–7 weeks of gestation. Of the men who successfully had sperm retrieved, 38/51 (74.5%) had surplus sperm cryopreserved beyond one cycle of IVF/ICSI (Table 2).

**Table 2:** Overall outcomes for men with NOA who underwent microTESE for IVF/ICSI (n = 72), expressed in means and standard deviations

Sperm retrieved at microTESE	51/72 (70.8%)
Fertilization with IVF/ICSI	31/43 (72.1%)
Reached ET	29/43 (67.4%)
Clinical pregnancy	21/29 (72.4%)
Live birth	18/29 (62.1%)
Surplus sperm cryopreserved	38/51 (74.5%)
Number of surplus cycles in those with surplus sperm	3.5 ± 1

Of the 38 men with surplus sperm cryopreserved, 20 (53%) of the couples achieved a pregnancy with live birth with the first IVF/ICSI cycle, 10 (26%) of them did not achieve a pregnancy with the first IVF cycle, and 8 (21%) of them had sperm cryopreserved for future use and have not undergone an IVF cycle yet or transferred sperm to another center. Of the 10 couples with surplus sperm available who did not achieve a pregnancy on the first IVF/ICSI cycle, 1 achieved a pregnancy with live birth on a second cycle, 2 have not undergone a second cycle, 2 had an additional negative IVF/ICSI cycle, 3 had an additional 2 negative cycles, and 2 had an additional 3 negative cycles including one with recurrent pregnancy losses with these cycles.

Overall, ten of the female partners were considered to have diminished ovarian reserve (DOR) defined as day 3 FSH of > 10 mIU/mL and/or antimüllerian hormone (AMH) < 1 ng/mL. The outcomes of couples with a female partner with DOR are described in Table 3. The mean age of the female partners was 32.7 ± 4.3 years of age. The mean age of female partners in couples in which microTESE/IVF/ICSI cycles resulted in live birth was 32.5 ± 3.7 years of age compared to 32.9 ± 4.5 years of age for the female partners for whom cycles did not result in live birth. There was not a statistically significant difference in mean female partner ages between the two groups (*p* = 0.845).

**Table 3:** Outcomes in couples with female partners with DOR (*n* = 10). * Patient 5 elected to not proceed with IVF/ICSI after microTESE

Patient	Sperm Retrieved	Fertilization with ICSI	Reach ET	Clinical Pregnancy	Live Birth	Donor Oocyte
1	Yes	Yes	Yes	No	No	No
2	Yes	Yes	Yes	Yes	Yes	Yes
3	Yes	Yes	Yes	No	No	Yes
4	Yes	No	No	No	No	No
5*	Yes	N/A	N/A	N/A	N/A	N/A
6	Yes	No	No	No	No	No
7	No	N/A	N/A	N/A	N/A	No
8	Yes	Yes	No	No	No	No
9	Yes	Yes	Yes	Yes	Yes	No
10	Yes	No	No	No	No	No

Although DOR status and age-related decline in female fertility are the primary obstacles from a female factor standpoint in these couples, 15 women in this cohort had other potential factors identified and managed medically or surgically prior to their IVF cycles. Six had endometrial polyps identified and hysteroscopically resected, 3 had polycystic ovarian syndrome (including 1 of the women who had a polyp as well), 1 had endometriosis, 4 had oligomenorrhea, 1 had a relevant uterine fibroid, and 1 had a history of previous unilateral oophorectomy. All of these conditions were treatable prior to, or with the IVF cycle and were not considered impactful to reproductive outcomes with IVF/ICSI with microTESE sperm.

### Multivariable modeling results

Table 4 presents the estimated odds ratios in our five multivariable logistic regression models. We did not identify any significant preoperative predictors of sperm retrieval, clinical pregnancy, or live birth (see Table 4). Male partner age was a significant predictor of reaching ET, with each year increase in age increasing the odds of reaching ET by 11% when adjusting for the other predictors (*p* = 0.049). FSH level had a marginally significant negative relationship with surplus sperm retrieved for more than one IVF/ICSI cycle. After adjusting for age, testicular volume, and FSH levels, men with a hypospermatogenesis histological pattern had significantly higher odds of sperm retrieval, clinical pregnancy, live birth, and having surplus sperm retrieved for more than one IVF/ICSI cycle. We note that for three of the five outcomes, only 64 cases were available for analysis due to missing values on the outcomes, further underscoring the significant relationships of the predictor variables identified in these cases.

**Table 4:** Estimates of adjusted odds ratios in multivariable logistic regression models for the five fertility outcomes analyzed in this study

Outcome	Sperm Retrieval	ET	Clinical Pregnancy	Live Birth	Surplus Sperm
Predictor Variable	*AOR (95% CI)*	*AOR (95% CI)*	*AOR (95% CI)*	*AOR (95% CI)*	*AOR (95% CI)*
Age (in years)	1.07 (0.98, 1.17)	1.11 (1.01, 1.23)*	1.04 (0.96, 1.13)	1.04 (0.95, 1.13)	1.04 (0.96, 1.12)
Testicular Volume	0.93 (0.83, 1.04)	0.97 (0.87, 1.09)	0.93 (0.83, 1.05)	0.94 (0.83, 1.06)	0.92 (0.82, 1.03)
FSH Level	0.98 (0.94, 1.02)	0.95 (0.90, 1.01)	0.96 (0.90, 1.02)	0.95 (0.89, 1.02)	0.96 (0.91, 1.01)
Testicular Histopathology					
*Maturation Arrest*	3.63 (0.36, 36.34)	2.32 (0.32, 16.88)	1.82 (0.25, 13.17)	0.86 (0.08, 9.53)	2.29 (0.40, 13.00)
*Hypospermatogenesis*	5.23 (1.27, 21.50)*	2.16 (0.58, 8.07)	5.13 (1.33, 19.71)*	4.85 (1.23, 19.15)*	7.50 (2.17, 25.97)*
*Sertoli Cell Only*	Ref	Ref	Ref	Ref	Ref
Sample Size	72	64	64	64	72

*AOR* Adjusted Odds Ratio. *CI* Confidence Interval. * denotes *p* < 0.05. Ref denotes reference category

## Discussion

Since the advent of microTESE for NOA due to primary testicular dysfunction, multiple investigators have assessed predictors such as age, testicular volume and FSH levels of various outcomes [7–20]. Most of this research has been focused on only the first step for the couple: retrieving sperm. Although this is a crucial first step to allow for successful pregnancies and live births downstream, to date there has been a paucity of investigation regarding predictors of the intermediate and conclusive steps, such as fertilization with IVF/ICSI, embryology progression to ET, clinical pregnancy, live birth, and having surplus sperm from the retrieval to allow for multiple IVF/ICSI cycles. To our knowledge, the current study is the most comprehensive assessment of the most numerous predictors for multiple milestones for these couples including the most critical, live birth, while accounting for male and female factors. It is also the only study evaluating predictors of surplus sperm cryopreserved for use beyond the initial IVF/ICSI cycle.

All patients in this cohort underwent varicocele repair if a palpable varicocele was present on their physical examination with reassessment of their semen analysis at 3 to 6 months post varicocele repair prior to undergoing microTESE. Men who had return of sperm to the ejaculate following varicocele repair did not undergo microTESE and were not included in this sample. Varicocele repair was recommended for all men with palpable varicoceles prior to microTESE as a number of studies have revealed return of sperm to the ejaculate in men with NOA who underwent varicocele repair [25–28]. There is data reporting that varicocele repair in men with NOA improves sperm retrieval rates at the time of microTESE and improves IVF/ICSI outcomes; therefore, the clinical protocol was to repair all palpable varicoceles and repeat semen analyses prior to offering microTESE [29–31].

Previously published studies on predictors of microTESE outcomes have primarily focused on sperm retrieval as the sole endpoint for success. In 2004, Tsujimura et al. found FSH, testosterone levels, and inhibin B levels to be the most influential predictive factors for sperm retrieval in a retrospective review of 100 men who underwent microTESE for NOA [7]. Ramasamy et al. published a retrospective study revealing that elevated FSH levels did not decrease the sperm retrieval rate with microTESE [8]. A retrospective review by Bernie et al. revealed that there is no individual clinical characteristic that accurately predicts sperm retrieval with microTESE in men with NOA [9]. A retrospective review by Bryson et al. reported that severely atrophic testicles did not impact the sperm retrieval rate in microTESE [10]. Ramasamy et al. performed a retrospective study which showed that age does not adversely impact sperm retrieval rates at the time of microTESE [11]. A retrospective study by Berookhim et al. demonstrated that men with NOA with Sertoli cell only histologic patterns, testicular volumes of ≥15 mL, and FSH between 10 and 15 mU/mL had poor sperm retrieval rates [12]. A meta-analysis in 2018 by Li et al. demonstrated that FSH, testicular volume, and testicular histopathology had limited predictive value for sperm retrieval [16]. The commonality of these previous studies is that the endpoint assessed for predictors was sperm retrieval at the time of microTESE in NOA men. Although sperm retrieval is a critical first step, downstream goals include fertilization, reaching ET, clinical pregnancy, and ultimately live birth for the couple. Surplus sperm beyond an initial IVF/ICSI cycle may be important for the couple as well.

This current study examined predictors for multiple microTESE/IVF/ICSI outcomes and revealed a hypospermatogenesis histology pattern as being significantly associated with several outcomes, including higher rates of sperm retrieval, clinical pregnancy, live birth, and having surplus sperm retrieved for future IVF/ICSI cycles when compared to maturation arrest or Sertoli cell only patterns. After adjusting for age, volume, and FSH levels, men with hypospermatogenesis patterns had 423% higher odds of having sperm retrieved. Those with maturation arrest histology also had substantially higher odds; however, this study was not powered to allow for detection of this odds ratio as significant. After adjusting for testicular volume, FSH, and histology pattern, each additional year of male age resulted in 11% higher odds of reaching ET. FSH levels trended towards significance with a negative relationship to odds of ET, but did not reach statistical significance. Adjusting for age, testicular volume, and FSH levels; those with hypospermatogenesis patterns had 413% higher odds of achieving a clinical pregnancy, 385% higher odds of live birth, and 650% higher odds of having surplus sperm retrieved for multiple IVF/ICSI cycles. FSH levels had a marginally significant negative relationship to retrieving surplus sperm. Although histopathology is not known in order to counsel couples prior to microTESE, it is useful to be able to counsel couples that the other potential predictors of outcomes should not be a deterrent to proceeding with microTESE/IVF/ICSI, even though they may be assumed to be unfavorable by patients and clinicians, they do not predict unfavorable outcomes.

An unexpected finding of this study was that male partner age was a significant predictor of reaching ET with older men having increased odds. This seems counterintuitive as there are data consistent with semen parameters and sperm DNA fragmentation worsening with increased age. However, the median age of the patient cohort in the current study was 35 years of age and only 15% (11/72) of them were in their forties, 2.8% (2/72) were in their fifties, and 2.8% (2/72) were in their sixties. Most of the men were in their twenties and thirties, so although age is a significant predictor, most men were below ages considered to be advanced paternal age which are typically the age ranges associated with poorer fertility outcomes overall. A previous publication revealed that increased age of men at the time of microTESE did not adversely impact sperm retrieval rates [11].

A limitation of this study is the sample size, although the statistical methodology utilized was appropriate for the size of the data set. The size of the sample is unique as NOA is present in only 1 % of men in the population. It would, therefore, be quite challenging for a single practicing reproductive urologist to accrue a larger sample of men undergoing microTESE, which would allow for more statistical power. Multi-site collaborations may be important to study this problem further in the future. An additional challenge to the power of the current study is that not all couples with NOA elect to undergo this level of treatment, technology, and expense.

A major strength and the novelty of this study is that all fertility diagnostic categories were included, making it the most comprehensive assessment of the predictors for multiple embryologic and clinical outcomes for these couples, including the most critical outcome, live birth, while accounting for male and female factors. It is also the only study evaluating predictors of surplus sperm cryopreserved for use beyond the initial IVF/ICSI cycle. The rate-limiting factor in the majority of these cases was NOA, based on overall female partner age, and there being no significant difference in mean female partner age between those for whom their cycles resulted in live birth versus those that did not. There was also only a small percentage of female partners in this cohort who were diagnosed with DOR, two of whom underwent donor oocyte cycles excluding oocyte quality as a variable in their outcomes.

## Conclusions

Men with NOA who underwent microTESE with a hypospermatogenesis testicular histopathology had better outcomes including rates of sperm retrieval, as well as downstream outcomes specifically clinical pregnancy, live birth, and having surplus sperm retrieved for more than one IVF/ICSI cycle, over those with histopathologic patterns of maturation arrest and Sertoli cell only. Increasing male partner age increased odds of reaching ET. No other clinical factors were significantly predictive for the outcomes considered in this study, which adds to the existing literature and aims to aid in the management and counseling of men with NOA.

## Data Availability

The datasets used are analyzed are during the current study are available from the corresponding author on reasonable request.

## Abbreviations

MicroTESE: microdissection testicular sperm extraction
NOA: non-obstructive azoospermia
IVF/ICSI: in-vitro fertilization/intracytoplasmic sperm injection
FSH: follicle stimulating hormone
ET: embryo transfer
DOR: diminished ovarian reserve
AMH: antimüllerian hormone
